# Antimicrobial potential of *Origanum vulgare* hydrolate on fresh produce: effect on rocket salad spiked with *Listeria monocytogenes*

**DOI:** 10.3389/fmicb.2026.1826527

**Published:** 2026-05-04

**Authors:** Francesco Buccioni, Chiara Purgatorio, Francesca Maggio, Stefania Garzoli, Chiara Rossi, Gabriella Centorotola, Francesco Pomilio, Annalisa Serio, Antonello Paparella

**Affiliations:** 1 Department of Bioscience and Technology for Food, Agriculture and Environment, University of Teramo, Teramo, Italy; 2 Department of Drug Chemistry and Technology, Sapienza University, Rome, Italy; 3 National Reference Laboratory for Listeria monocytogenes, Istituto Zooprofilattico Sperimentale dell’Abruzzo e del Molise G. Caporale, Teramo, Italy

**Keywords:** food safety, hydrolate, *Listeria monocytogenes*, *Origanum vulgare*, rocket salad, washing formulation

## Abstract

**Introduction:**

Fresh-cut salads are increasingly recognized as possible carriers of foodborne pathogens, including *Listeria monocytogenes* (*L. monocytogenes*). As interest in their consumption rises, maintaining their microbiological safety is crucial. To this effort, this study aimed to evaluate *Origanum vulgare* (*O. vulgare*) subsp. *Hirtum* hydrolate as an antimicrobial washing solution for rocket salad spiked with the pathogen *L. monocytogenes*.

**Material and methods:**

First, *in vitro* investigations were performed to assess the Minimum Inhibitory Concentration (MIC) of the hydrolate against three *L. monocytogenes* strains and analyze their growth potential at incremental hydrolate concentrations. Subsequently, the hydrolate was applied as a dipping solution to rocket salad (500 μL mL^−1^, 5 min) that was then stored at 4 °C for 48 h. Enumeration of the inoculated *L. monocytogenes*, as well as of the rocket salad microbiota, were performed. Physical–chemical (pH, a_w_, and color) and sensory characteristics were also evaluated.

**Results:**

The treated samples showed a reduction of *L. monocytogenes* load (about 1.0 log CFU/g) and a modest effect on total mesophilic and psychrophilic counts, *Bacillus* spp., and lactic acid bacteria, compared with control samples washed with Phosphate-Buffered Saline solution (PBS). As a positive result, the treatment did not affect significantly the physical–chemical and sensory characteristics of the rocket salad during storage.

**Discussion:**

The treatment demonstrated the antimicrobial potential of *O. vulgare* against *L. monocytogenes* while preserving product quality; however, further optimization is required to enhance effectiveness, particularly against contaminant microbiota.

## 1 Introduction

Fruits and vegetables are widely recognized as healthy foods and are frequently associated with reduced risk of cardiovascular diseases. In fact, these foods are demonstrated to play a positive role in the case of cancer, atherosclerosis, and diabetes, which are responsible for more than 70% of deaths worldwide (Lapuente et al., 2019). The biological activity of these foods is mainly attributed to their valuable content in dietary fiber, vitamins, minerals, and abundant phytochemicals (Jideani et al., 2021). Based on that, the updated recommendations for vegetable intake increased the previous 240 g per day to 300 g per day (Willett et al., 2019).

Rocket salad is a leafy vegetable belonging to the Brassicaceae family, mainly including plants of the *Eruca sativa* species, characterized by a bitter, pungent flavor. This vegetable is appreciated for its bioactive compounds, including glucosinolates, quercetin, kaempferol, and vitamin C (Martínez-Sánchez et al., 2006). Rocket is mostly consumed as a fresh-cut product, either alone or as an ingredient in mixed salads or sandwiches (Cefola and Pace, 2015). In fact, the frenetic lifestyle of people has increased the demand for ready-to-eat (RTE) products, which are easier to prepare than fresh vegetables, which instead require several preliminary preparation steps such as washing, cutting, and seasoning. However, fresh-cut rocket salad poses significant concerns for food safety due to its high water content, permissive pH range (6.0–7.0), lack of decontamination treatments, and the possibility of undergoing temperature abuse during processing, transport, and storage operations. Therefore, implementing effective strategies to control potentially harmful microorganisms is critical for this product.

*Listeria monocytogenes* (*L. monocytogenes*) is a psychrotrophic and halotolerant bacterium responsible for listeriosis, a food-related illness particularly dangerous for the YOPI (young, old, pregnant, and immunocompromised) categories (Cherifi et al., 2020). Many listeriosis outbreaks associated with RTE vegetables have been recorded, such as the ones caused by packaged salads in the United States, which led to an alarming number of deaths in the last decade (CDC, 2016; Zhu et al., 2017; CDC, 2021). Therefore, according to the literature, *L. monocytogenes* is one of the major pathogens contributing to foodborne outbreaks associated with fresh-cut vegetable products. In fact, recent studies have shown the prevalence of this microorganism in fresh-cut processing facilities (Gil et al., 2024), with 30% of the analyzed tests (environments after processing, before cleaning and disinfection) resulting in positive results. Among these, the most contaminated were those processing fresh-cut salads, followed by fresh-cut vegetables and fresh-cut fruit. Furthermore, the high prevalence of *L. monocytogenes* has been reported in fresh-cut fruits and vegetables, and, among them, leafy vegetables displayed the highest incidence, also due to the storage at refrigeration temperatures at which this pathogen can easily survive and develop (Silva et al., 2017; Zhang et al., 2020). This rapidly growing interest in adopting new strategies to improve consumer safety.

To date, the only critical control point for *L. monocytogenes* hazard in fresh-cut vegetables is the washing step (Giang et al., 2022). Nevertheless, the washing procedures have some side effects, such as being highly dependent on leaf shape and texture, and are usually carried out with chlorinated water (Nastou et al., 2012). In recent years, evidence of the toxicity of chlorinated substances has grown, along with knowledge of the development of resistance by pathogenic microorganisms, including *L. monocytogenes*, due to its abuse in the food industry (Poimenidou et al., 2016). These issues have led to renewed interest in innovative, more sustainable alternatives to synthetic food preservatives. Moreover, consumers became more careful about food formulations, perceiving the foods with natural additives as healthier.

Within natural substances with preservative potential, essential oils (EOs) are the most studied because of their strong activity even at low concentrations. However, their hydrophobicity and strong flavor pose some limitations to their use. To overcome these problems, the use of hydrolates (HYs) is gaining ground in research. HYs are by-products of the distillation process of EOs (Rossi et al., 2022). They consist of aqueous solutions, in which a small oily fraction of active molecules deriving from the corresponding EOs is dispersed. Consequently, they have a lower sensory profile than EOs, are cheaper and more hydrophilic, making them more suitable for food applications (D’Amato et al., 2018). Although studies on HYs are increasing, research remains limited compared to EOs, particularly regarding *in situ* studies.

*Origanum vulgare* (*O. vulgare*) subsp. *hirtum* EO showed a very interesting activity, causing stress to *L. monocytogenes* cells, and restoring its antibiotic susceptibility (Maggio et al., 2022). For this reason, it can be hypothesized that the corresponding hydrolate (OHY) may also be effective against *L. monocytogenes*. In fact, few studies on the antimicrobial activity of OHY are reported in the literature (Saǧdιç, 2003; Gülmez et al., 2006; Ozturk et al., 2016), whereas numerous studies demonstrated how the corresponding EO and carvacrol (the main phenolic compound present in oregano EO) tested individually show their antimicrobial activity *in situ* (Paparella et al., 2016; Silva et al., 2017; Kraśniewska et al., 2020; Hajibonabi et al., 2023; Lastra-Vargas et al., 2023; Mączka et al., 2023; Pinto et al., 2024).

Starting from these data, our research aims to investigate the potential application of OHY as a natural alternative washing solution potentially applicable during the production process of fresh-cut rocket, but also in the domestic environment, to counter the hazard of *L. monocytogenes* and to control the microbial groups responsible for spoilage while maintaining the physical–chemical and sensorial quality of the product.

## 2 Materials and methods

### 2.1 Hydrolate

The commercial, food-grade OHY, kindly provided by Exentiae S.r.l. (Catania, Italy), was used in this study. The OHY was stored at 4 °C in a dark bottle until analysis. For the tests, OHY was diluted in 10 mM Phosphate-Buffered Saline (PBS) (pH 7.4) in different concentrations depending on the analysis.

### 2.2 Bacterial strains and inocula preparation

The study was performed by using three different *L. monocytogenes* strains to increase the variability of the inoculum, given differences in growth and survival among different strains (Spanu et al., 2014). *L. monocytogenes* type strain ATCC 7644 (serogroup 1/2c), and the strains *L. monocytogenes* 2, isolated from lettuce (serogroup 4b, clonal complex CC6), and *L. monocytogenes* 4, isolated from spinach (serogroup 2a, clonal complex CC14), which were previously isolated, identified, and characterized by the Istituto Zooprofilattico Sperimentale dell’Abruzzo e del Molise (IZSAM), were used in both *in vitro* and *in situ* analyses. Strains were stored at −80 °C in Brain Heart Infusion (BHI) enriched with 20% glycerol and were routinely cultivated on BHI broth (Liofilchem S.r.l., Roseto degli Abruzzi, Italy) and BHI agar plates. Before each analysis, one *L. monocytogenes* colony from each strain was transferred to BHI broth and incubated overnight at 37 °C to obtain a fresh working culture at the beginning of the stationary phase. Bacterial cells were sequentially harvested by centrifugation at 15900 RCF x g for 5 min using a 5415D centrifuge (Eppendorf, Hauppauge, NY, United States) and washed twice with PBS, as described by Paparella et al. (2016). Then, inocula were standardized to 8 log CFU mL^−1^ by absorbance measurements at OD_600_ (Jenway 6305 UV/Vis spectrophotometer, Bibby Scientific, Ltd., Stone, UK).

### 2.3 Rocket salad

Fresh rocket salad (*Eruca sativa*) was purchased from a local retailer, packed in a recycled polyethylene terephthalate tray and a polyethylene bag in air, the same day as the first analysis, and stored at 4 °C.

### 2.4 Head space GC–MS and GC-FID analysis

The volatile chemical profile of OHY was characterized using a Headspace Turbomatrix 40 (PerkinElmer, Waltham, MA, United States) autosampler connected to a gas chromatograph equipped with a flame ionization detector (FID) directly coupled to a mass spectrometer (Clarus 500 model, PerkinElmer, Waltham, MA, United States). Approximately 2 mL of hydrolate was placed in a 20 mL vial sealed with a headspace PTFE-coated silicone rubber septum and cap.

The chromatographic separation of the detected components was performed with a Varian Factor Four VF-1 capillary column. Helium was used as a carrier gas at a constant flow of 1 mL/min. The mass spectrometer was equipped with an ion source (EI) set to 70 eV; the mass spectra were scanned in the range 40–500 m/z. The temperature of the ion source and the connection parts were 200 °C and 220 °C, respectively.

The oven temperature program was as follows: from 60 °C ramped to 220 °C at a rate of 6 °C min^−1^, and finally isothermal at 220 °C for 15 min. The identification of components was performed by matching their mass spectra to those stored in the NIST 11 mass spectral library database. Furthermore, the linear retention indices (LRIs) were calculated using a series of alkane standards and compared with available retention data reported in the literature. For quantitative determination, the analysis was also conducted through a Clarus 500 GC series GC-FID. The peak areas of the FID signal were used to calculate the relative concentrations of the components expressed as percentages without the use of an internal standard or any factor correction. All analyses were carried out in triplicate.

### 2.5 Determination of minimum inhibitory concentration and growth kinetics

MIC of OHY was evaluated by the microdilution method in a 96-well microtiter plate (Corning Inc., Kennebunk, ME, United States) for each *L. monocytogenes* strain separately. The initial bacterial suspensions were then diluted in PBS to obtain a 7 log CFU mL^−1^ inoculum. Microtiter plates were then inoculated according to CLSI standards (CLSI, 2020). Growth kinetics were obtained using an Omnilog reader (Biolog Inc., Hayward, CA, United States), which constantly monitored bacterial growth via optical density (OD_590_) measurements every 15 min. MIC values were defined as the concentrations at which no growth was observed. Uninoculated medium (Mueller-Hinton Broth, Liofilchem S.r.l., Roseto degli Abruzzi, Italy) was used as a blank for measurements. The analysis was conducted in five replicates and expressed as the mean of the repetitions.

### 2.6 Samples preparation for *in situ* analysis

**Figure 1:**
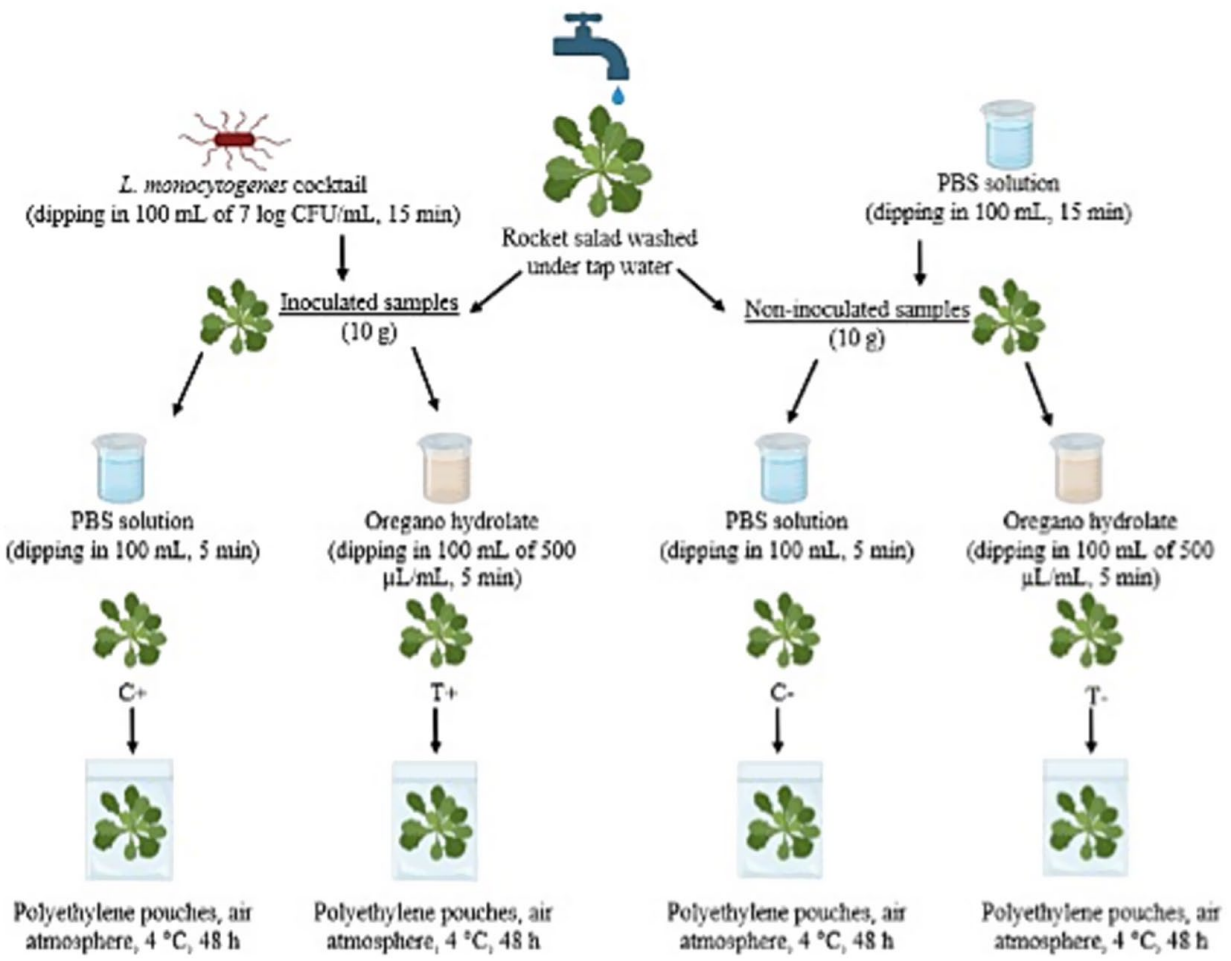
Processing flowchart of the experimental design (created in Biorender.com).

After preculture and recovery of the inocula, the bacterial suspensions were diluted with PBS to obtain a 7 log CFU mL^−1^ inoculum. At the end, the three different inocula were mixed to obtain a cocktail of strains with a final load of 7 log CFU mL^−1^. Figure 1 describes the processing flowchart of the experiment. The purchased rocket salad was initially washed under tap water. At this point, samples were divided into inoculated and non-inoculated samples. Non-inoculated samples were used to conduct physical-chemical and sensory analyses and to evaluate whether the *L. monocytogenes* inoculum could influence other naturally occurring microbial groups in rocket, as well as the OHY antimicrobial effect. Inoculated control samples (positive control C+) and non-inoculated control samples (negative control C−) were not submitted to OHY treatment, whereas inoculated treated samples (positive treated T+) and non-inoculated treated samples (negative treated T−) were treated with OHY. The inoculating procedure was performed as follows: for inoculated samples, 10 g of rocket salad leaves were dipped in 100 mL of an *L. monocytogenes* suspension for 15 min, then left to dry for 45 min on sterile gauze under laminar flow in a BSC II cabinet (C+ and T+). For non-inoculated samples (C− and T−), 10 g of rocket leaves were dipped in 100 mL PBS solution for 15 min and subsequently left to dry for 45 min, as for inoculated samples. After the inoculation procedure, treated samples were dipped in 100 mL of 500 μL mL^−1^ OHY solution for 5 min (T+ and T−), whereas control samples were dipped in 100 mL of sterile PBS for 5 min (C+ and C−); both were left to dry as previously described. All the samples were sealed in polyethylene pouches (air atmosphere) and kept at refrigeration temperature (4 °C) for 48 h. Each sample was prepared in triplicate. Other aliquots of 100 g each of the C− and T− samples were prepared to perform physical–chemical and sensory analyses. The experiment was repeated three times. Microbiological samples were analyzed immediately after the treatment (T0), then after 24 h (T24), and after 48 h (T48) of storage at 4 °C. For microbiological analysis, 10 g of each sample was transferred aseptically to a Stomacher bag and homogenized with 1:10 PBS for 90 s at 260 rpm using a lab-scale homogenizer (Lab-blender 400 Circulator, Seward, Worthing, UK). The resulting suspension was then 10-fold diluted for the enumeration of microbial loads, using the following media, all from Liofilchem S.r.l. (Roseto degli Abruzzi, Italy): Plate Count Agar (PCA) for total mesophilic and psychrophilic counts (TMC and TPC, respectively), incubated aerobically at 30 °C and 4 °C for 48 h and 7 days, respectively; *Pseudomonas* Agar Base (PSA) for *Pseudomonas* spp., incubated at 25 °C for 48 h; PEMBA (*Bacillus cereus* Agar Base) for *Bacillus* spp. count, incubated at 30 °C for 48 h; Yeast Peptone Dextrose agar with 150 ppm Chloramphenicol (YPD) for total yeasts and molds count, incubated at 25 °C for 7 days; De Man, Rogosa, and Sharpe (MRS) agar for lactic acid bacteria (LAB) count, incubated at 30 °C for 72 h; Violet Red Bile Glucose Agar (VRBGA) for Enterobacteriaceae, incubated at 37 °C for 48 h. *L. monocytogenes* enumeration was performed by aseptically transferring 10 g of each sample into stomacher bags and homogenizing with 1:10 Half Fraser Broth (Oxoid, Ltd., Basingstoke, Hampshire, UK). Tenfold dilutions were prepared for enumeration on Agar *Listeria* Ottaviani & Agosti (Biolife Italiana S.r.l., Milan, Italy) and incubated at 37 °C for 48 h in accordance with ISO 11290-2:2017 (ISO, 2017). Incubation was carried out in aerobic conditions. Two replications for each plated dilution were performed. All the experiments were performed in triplicate. Results were expressed as log CFU g^−1^ ± standard deviation.

### 2.7 Physical–chemical analyses

Analyses of pH, a_w_, and color were performed at T0, T24, and T48 on C− and T− samples.

The pH was measured using a pH meter (Mettler Toledo MP 220, Novate Milanese, Italy). Aliquots of 10 g of rocket leaves were homogenized in 1:1 PBS, and the pH was determined. Eight replications were performed for each sample.

The a_w_ of the samples was measured using an Aqualab 4TE (METER Group, Inc., Hopkins, NE, United States). Homogeneous samples were transferred into disposable cups, with the bottom of each cup covered to obtain an accurate reading. Eight replications were performed for each sample.

Color analysis was carried out, recording CIELab color space coordinates using a colorimeter (Colorimeter CR-5, Konica Minolta Sensing Europe, Tokyo, Japan) equipped with a CM-A195 3 mm target mask. Values were obtained via reflectance of the samples. Results reported are the mean values of 30 leaves from each sample. The value ΔE (global color difference) was also evaluated, according to Paparella et al. (2016).

### 2.8 Sensory analyses

Finally, a sensory analysis was conducted on C− and T− samples to evaluate the sensory impact of the OHY treatment on the rocket salad. The analysis was performed by evaluating the odor, color, aroma, size, shape, and overall acceptability of rocket salad leaves. Twenty healthy, nonsmoking, not color-blind, trained panelists were selected and submitted each to the Triangle test (ISO 4120:2004) (ISO, 2004) and consumer acceptability testing. All participants were recruited voluntarily and provided informed consent before the sensory test. For the Triangle test, two identical white dishes were submitted to panelists, divided into three sections. In the first dish, three leaves were placed, occupying two sections for control and one section for the treated sample. In the second trial, three leaves were placed, occupying two sections for treated and one section for control samples. Panelists were asked to identify the odd sample and define their willingness to buy it. An open-ended section was introduced to allow panelists to explain how the selected sample differed from the others, and a discrete scale (from 1 = minimum acceptability to 5 = maximum acceptability) was added to assess their acceptance of the single samples. The panel test was conducted in a sensory evaluation laboratory equipped with a temperature-controlled room, with samples served at room temperature (20 °C).

### 2.9 Data analysis

Data values are reported as the mean with standard deviation. A two-way ANOVA followed by Tukey’s multiple comparisons test was performed using XLSTAT statistical software (*p < 0.05) to determine statistically significant differences among results.

Selected growth kinetics obtained from the Omnilog reader were processed by fitting them to the Gompertz model equation reparametrized by Zwietering et al. (1990).

Statistical significance for the Triangle Test was evaluated using unilateral *p* = 1/3 significance tables (Paparella et al., 2016).

## 3 Results

### 3.1 Chemical composition

By HS-GC/MS and GC/FID analysis, six volatile compounds were identified and quantified in the OHY (Table 1). Carvacrol was the main component (95.9%), followed by a small amount of thymol (2.9%). The other components identified in the vapor phase ranged from 0.1 to 0.4%.

**Table 1:** Chemical volatile composition (percentage mean value ± standard deviation) of OHY, as determined by HS-SPME/GC–MS.

No	Component^a^	LRI^b^	LRI^c^	Hydrolate (%)
1	1-octen-3-ol	980	983	0.4 ± 0.02
2	p-cymene	1,085	1,091	0.1 ± 0.00
3	linalool	1,091	1,095	0.2 ± 0.01
4	terpinen-4-ol	1,178	1,182	0.4 ± 0.02
5	thymol	1,271	1,279	2.9 ± 0.03
6	carvacrol	1,300	1,304	95.9 ± 0.09
	SUM			99.9

a The components are reported according to their elution order on apolar column.

b Linear Retention Indices measured on apolar column.

c Linear Retention Indices from literature.

### 3.2 Determination of minimum inhibitory concentration and growth kinetics

*L. monocytogenes* cells were exposed to incremental concentrations of OHY and incubated for 48 h at 37 °C in the Omnilog reader. After incubation, the resulting data were elaborated to obtain the growth parameters of the three strains. As reported in Table 2, the treatment with 500 μL mL^−1^ OHY had an inhibitory effect on all *L. monocytogenes* strains, where no visible growth was observed.

**Table 2:** Growth parameters of the three *L. monocytogenes* strains exposed to increasing OHY concentrations, obtained by fitting the curves according to the Gompertz equation reparametrized by Zwietering et al. (1990).

Strain	OHY (μL mL^−1^)	Lag phase (h)	Maximum rate (OD_600_^h−1^)	Maximum growth value^a^ (OD_600_)	*R* ^2^	Model^b^
*L.m.* ATCC7644	0 (Ctrl)	1.704 ± 0.126	0.0126 ± 0.0014	0.169 ± 0.021	0.994	Complete
	125	2.242 ± 0.816	0.0130 ± 0.0030	0.200 ± 0.008	0.983	Complete
	250	3.436 ± 0.345	0.0121 ± 0.0008	0.150 ± 0.022	0.949	Complete
	500	–	–	–	–	Unmodelable
*L.m.* 2	0 (Ctrl)	8.758 ± 1.182	0.0096 ± 0.0000	0.147 ± 0.004	0.998	Complete
	125	10.625 ± 0.347	0.0110 ± 0.0011	0.147 ± 0.029	0.996	Complete
	250	11.511 ± 0.644	0.0082 ± 0.0010	0.158 ± 0.008	0.999	Complete
	500	–	–	–	–	Unmodelable
*L.m.* 4	0 (Ctrl)	14.126 ± 0.634	0.01145 ± 0.0060	0.194 ± 0.028	0.998	Complete
	125	17.639 ± 2.307	0.01411 ± 0.0007	0.171 ± 0.007	0.999	Complete
	250	5.033 ± 4.822	0.0017 ± 0.000	0.114 ± 0.005	0.983	No asymptote
	500	–	–	–	–	Unmodelable

a Final cell density value.

b Assessment of the fitting of the model.

However, at lower concentrations, the OHY effect was different in the wild strains under investigation: particularly, whereas a concentration of 250 μL mL^−1^ could extend to roughly 12 h the lag phase of *L. monocytogenes* 2, at the same concentration *L. monocytogenes* 4 showed a much shorter lag phase (5 h), with a reduced maximum growth value. ATCC 7644 was less affected, particularly in the lag phase extension. Notably, OHY demonstrates the potential to reduce the final concentration of *L. monocytogenes* in almost all concentrations investigated and the antimicrobial effect on the growth dynamics.

### 3.3 Microbiological analysis of rocket salad

Considering the results obtained by treating with the *L. monocytogenes* strains, 500 μL mL^−1^ OHY was used as a washing solution for rocket salad. The counts of all microbial groups for all rocket salad samples over time are reported in Table 3.

**Table 3:** Microbial load of TMC, TPC, inoculated *L. monocytogenes*, *Bacillus* spp., LAB, *Enterobacteriaceae*, *Pseudomonas* spp., yeasts and molds, in rocket salad stored at 4°C for 48 h.

Microbial group/Sample	C+	T+	C−	T−
TMC				
0 h	6.67 ± 0.13 a, B	6.10 ± 0.10 b, C	6.54 ± 0.09 a, C	6.01 ± 0.03 b, B
24 h	6.86 ± 0.02 a, B	6.34 ± 0.03 b, B	6.38 ± 0.03 b, C	5.86 ± 0.05 c, C
48 h	7.28 ± 0.05 a, A	6.95 ± 0.06 b, A	7.34 ± 0.01 a, A	6.71 ± 0.04 c, A
TPC				
0 h	6.34 ± 0.06 a, A	5.71 ± 0.12 b, A	5.78 ± 0.11 b, A	5.08 ± 0.18 c, A
24 h	6.00 ± 0.05 a, B	5.69 ± 0.08 b, AB	5.79 ± 0.12 b, A	5.14 ± 0.11 c, A
48 h	5.81 ± 0.06 a, C	5.52 ± 0.07 a, B	5.58 ± 0.06 a, B	4.51 ± 0.45 b, B
*Listeria monocytogenes*				
0 h	5.57 ± 0.02 a, C	4.72 ± 0.03 b, C	Not detected	Not detected
24 h	5.88 ± 0.04 a, B	4.95 ± 0.05 b, B	Not detected	Not detected
48 h	6.50 ± 0.01 a, A	5.68 ± 0.10 b, A	Not detected	Not detected
*Bacillus* spp.				
0 h	6.41 ± 0.01 a, A	5.77 ± 0.02 c, B	6.29 ± 0.05 b, B	5.74 ± 0.03 c, A
24 h	6.41 ± 0.07 a, A	5.75 ± 0.02 b, B	6.35 ± 0.04 a, A	5.48 ± 0.01 c, B
48 h	6.41 ± 0.03 a, A	6.21 ± 0.09 b, A	6.25 ± 0.02 b, B	5.72 ± 0.01 c, A
LAB				
0 h	6.40 ± 0.06 a, B	6.04 ± 0.06 b, B	6.42 ± 0.12 a, B	6.02 ± 0.10 b, A
24 h	6.68 ± 0.07 a, A	6.25 ± 0.10 b, A	6.60 ± 0.08 a, A	6.08 ± 0.08 b, A
48 h	6.02 ± 0.01 a, C	5.34 ± 0.06 c, C	5.47 ± 0.05 b, C	5.26 ± 0.03 d, B
*Enterobacteriaceae*				
0 h	6.69 ± 0.01 a, A	6.35 ± 0.07 b, A	6.75 ± 0.02 a, A	6.21 ± 0.13 c, B
24 h	6.73 ± 0.05 a, A	6.39 ± 0.08 c, A	6.64 ± 0.01 b, B	6.36 ± 0.03 c, A
48 h	6.62 ± 0.05 a, B	6.37 ± 0.03 b, A	6.60 ± 0.03 a, C	6.29 ± 0.05 c, AB
*Pseudomonas* spp.				
0 h	6.37 ± 0.09 a, C	6.42 ± 0.04 ab, B	6.55 ± 0.04 b, A	6.56 ± 0.03 b, A
24 h	6.97 ± 0.09 a, A	6.48 ± 0.02 b, C	6.99 ± 0.02 a, A	6.74 ± 0.04 a, B
48 h	6.81 ± 0.02 a, B	6.41 ± 0.05 c, B	6.64 ± 0.26 b, A	6.31 ± 0.02 d, B
Yeasts and molds				
0 h	2.75 ± 0.18 ab, B	2.75 ± 0.18 a, B	3.03 ± 0.26 a, A	2.31 ± 0.23 b, B
24 h	3.11 ± 0.26 a, AB	2.90 ± 0.16 ab, AB	2.82 ± 0.26 ab, A	2.66 ± 0.13 b, A
48 h	3.19 ± 0.15 a, A	3.08 ± 0.16 a, A	3.08 ± 0.20 a, A	2.62 ± 0.15 b, AB

A significant difference (*p* < 0.05) for different samples at the same time is represented by different lowercase letters along the same row, whereas for the same sample at different times, by different uppercase letters along the same column. Results are expressed as log CFU g^−1^. C+: inoculated untreated samples (positive control); T+: inoculated treated samples; C−: not-inoculated untreated samples (negative control); T−: not-inoculated treated samples.

As expected, non-inoculated samples (C−) confirmed the absence of *L. monocytogenes* in the salad. Instead, the inoculated *L. monocytogenes* load was around 5.5 log CFU g^−1^ for the C+ samples at T0. At T0, treatment with 500 μL mL^−1^ OHY determined a significant reduction of 0.85 log CFU g^−1^ of the *L. monocytogenes* load (samples T+), showing an anti-listeria activity at the early stages of the treatment. This activity was even more evident after 24 h, when the reduction was about 1 log CFU g^−1^. Forty-eight hours after treatment, the reduction was still significant, and the cell load was comparable with that observed at T0 without any treatment (C+). Therefore, OHY appeared to exert an immediate antilisterial activity, which was maintained throughout refrigerated storage.

Regarding the TMC, significant differences were observed in both positive and negative samples. In fact, the fresh rocket salad used in this study is characterized by a complex microbiota, with the TMC of 6.54 log CFU g^−1^ at T0, consistent with loads commonly reported for commercially available leafy greens (Martínez-Sánchez et al., 2006; Zhang et al., 2020). C+ and C− loads were similar, as well as the reductions observed for T+ and T−, respectively. At T0, the load reduction was approximately 0.5 log CFU g^−1^, and it remained generally stable until 24 h. At T48, the reduction decreased for positive samples while slightly increased for negative samples (0.63 log CFU g^−1^ reduction), suggesting that in not-inoculated samples, with lower microbial loads, OHY could be more effective.

As regards the TPC, a reduction in the count difference over time was revealed between C+ and T+, going from 0.63 at T0 to 0.29 at T48. Instead, in not-inoculated samples, the difference remained almost constant until T24 and increased to 1.02 log CFU g^−1^ at T48, when the count of T− dropped to 4.52 log CFU g^−1^, in comparison with 5.58 log CFU g^−1^ of C−. This behavior could be explained by differences in initial loads: in fact, it has been demonstrated that higher microbial loads make it more difficult to achieve a reduction with several antimicrobials. In our case, this is even more true due to the psychrophilic nature of *L. monocytogenes*, which can compete with other resistant bacteria (Cherifi et al., 2020).

Presumptive *Bacillus* spp. counts followed a similar trend in both positive and negative samples, increasing from T0 to T24 and decreasing from T24 to T48; however, in this case, the reduction in negative samples was more evident, likely for the previous hypothesized reasons. The major difference was observed at T24, where a 0.66 log CFU g^−1^ reduction among C+ and T+, and 0.87 between C− and T− was observed.

Effectiveness against LAB was variable; it was similar between positive and negative samples until T24 (around 0.5 log CFU g^−1^ reduction), whereas it was slightly more accentuated for positive (0.68 log CFU g^−1^ reduction) than for negative (0.21 log CFU g^−1^ reduction) samples at T48.

Presumptive *Pseudomonas* spp. only showed a slight reduction at T24 and T48 in all the samples (maximum reduction of 0.4 log CFU g^−1^). For yeasts and molds, the susceptibility to OHY was very low and generally not significant. However, yeast and mold loads were generally below 3 log CFU g^−1^, confirming that these microbial groups are not relevant to the shelf life of fresh vegetable spoilage (Alegbeleye et al., 2022). As regards *Enterobacteriaceae*, although the reduction was always significant, a decrease higher than 0.5 log CFU g^−1^ was observed only for negative samples at T0.

### 3.4 Physical–chemical analyses

The pH values were stable over time for both C− and T− samples, with treated samples showing lower pH at each time, due to the hydrolate treatment. In fact, the pH of the OHY washing solution was 3.85 ± 0.03, which could cause a reduction in pH in the T− sample. Nevertheless, the difference between control and treated samples was not significant (Table 4).

**Table 4:** pH and a_w_ values in rocket salad stored at 4 °C for 48 h.

Sample	pH		a_w_	
	C−	T−	C−	T−
0 h	6.24 ± 0.02 a, A	6.12 ± 0.32 a, A	0.986 ± 0.004 a, A	0.984 ± 0.003 a, A
24 h	6.21 ± 0.08 a, A	6.13 ± 0.04 a, A	0.984 ± 0.008 a, A	0.980 ± 0.008 a, A
48 h	6.24 ± 0.17 a, A	6.16 ± 0.05 a, A	0.976 ± 0.015 a, A	0.980 ± 0.011 a, A

A significant difference (*p* < 0.05) for different samples at the same time is represented by different lowercase letters along the same row, whereas for the same sample at different times, by different uppercase letters along the same column. C−: not-inoculated untreated samples (negative control); T−: not-inoculated treated samples.

The a_w_ values were very similar both among C− and T− samples and over time, without any significant variation (Table 4).

**Figure 2:**
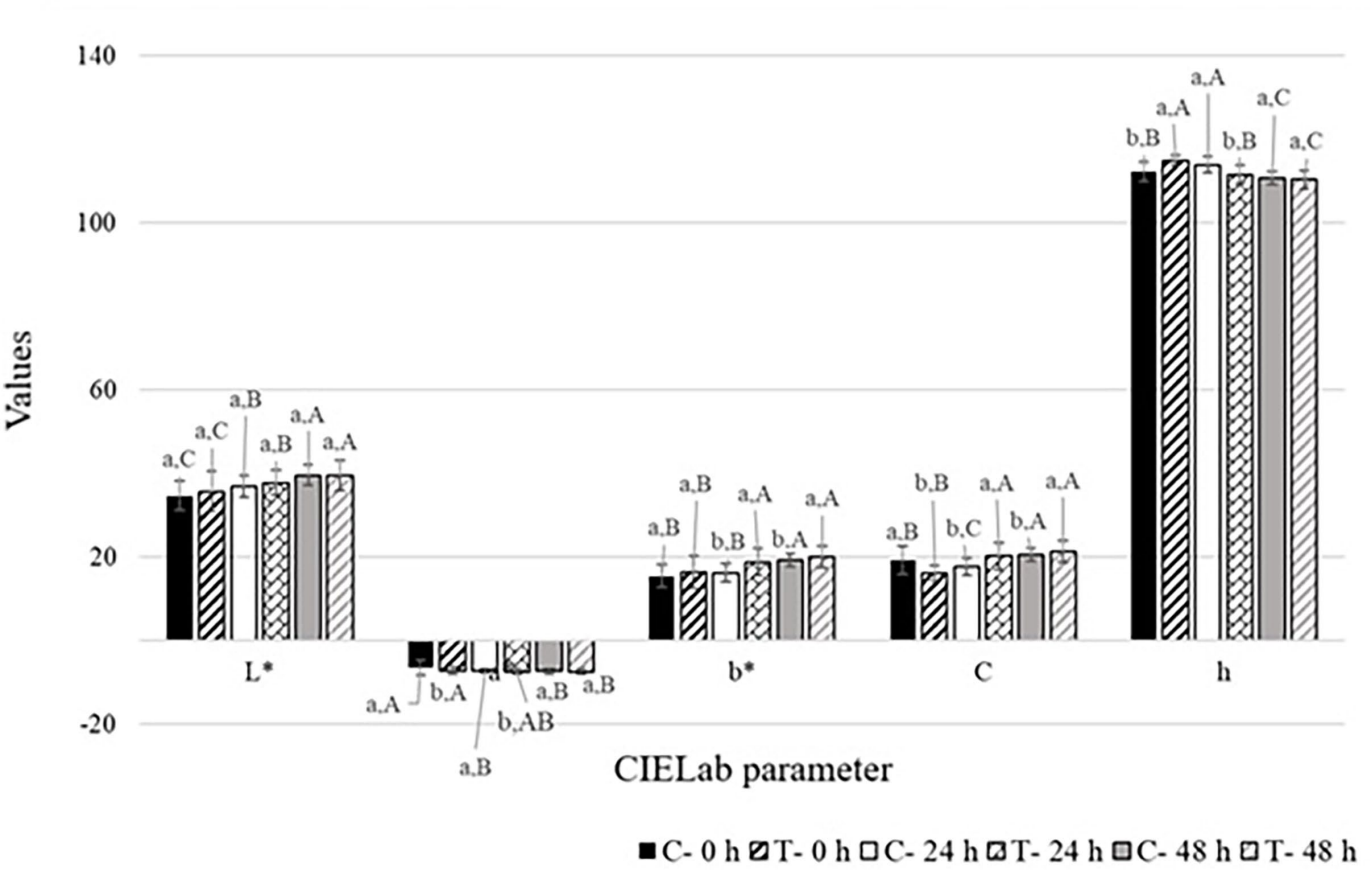
L*, a*, b*, C*, *h* values of rocket salad stored at 4 °C for 48 h. Significant differences (*p* < 0.05) for different samples at the same time are represented by different lowercase letters, whereas for the same sample at different times, by different uppercase letters. C−: not-inoculated control sample, T−: not-inoculated treated sample.

CIELab color parameters (L*, a*, b*, C, and h) were evaluated to assess whether OHY could determine a color change and, therefore, alter the consumers’ perception of the food (Figure 2). L* indicates the lightness of the samples for higher values. No significant differences were noticed between control and treated samples at any of the analysis times. In general, an increase in L* value was observed over time, indicating a qualitative deterioration linked to the discoloration of the leaves, which are therefore detected as lighter by the instrument. The value of a* indicates an increasingly green trend as it moved toward negative values. *T*-value was significantly lower at T0 and T24, suggesting a protection of the green color of the leaves, although the values balanced with those of the C− sample at T48. The b* values represent a tendency toward yellow for higher values. Values were comparable at T0, and both C− and T− samples tended to increase over time, with a greater increase in T− than in C− samples. Saturation (C*) was significantly lower for T− than C− samples at T0, but the values became more similar at the following times. Hue variation (h) was also more different at T0 (significantly higher for T− than C−), then balancing at T48. Finally, the ΔE value was evaluated because this parameter measures the overall variation of the color and is the most useful for assessing whether the hydrolate had any effects that are relevant and perceptible by consumers on the rocket leaves. ΔE was 5.31 at T0, decreased to 4.02 at T24, and declined again to 3.41 at T48. These values indicated that global color difference decreased, with samples becoming increasingly similar over time.

### 3.5 Sensory analyses

Triangle Test (Table 5) stated that the odd sample was recognized as different a significant number of times (34 out of 40) at T0, but not at T24 and T48 (14 and 11 times out of 40, respectively). This is most likely due to the loss of the sensory notes of the hydrolate over time. In agreement, the consumers’ judgments described the treated sample at T0 as having a “more pungent,” “penetrating,” and “persistent odor” compared to the control, which was instead described as having a “lighter” and “fresher” aroma. At T24, in one case, T− was described as “slightly more aromatic” than C−, whereas at T48, no judgment was made regarding the relevant difference between the two samples, as OHY lost its characteristic flavor. Color was described as very similar between the two samples at all times, except for one panelist, who described the C− as “clearer” than the control.

**Table 5:** Triangle test of C− vs. T−.

Time	N. Responses	Correct responses	Significance
0 h	40	34	*p* < 0.05
24 h	40	14	*p* > 0.05
48 h	40	11	*p* > 0.05

C−: not-inoculated control sample; T−: not-inoculated treated sample.

**Figure 3:**
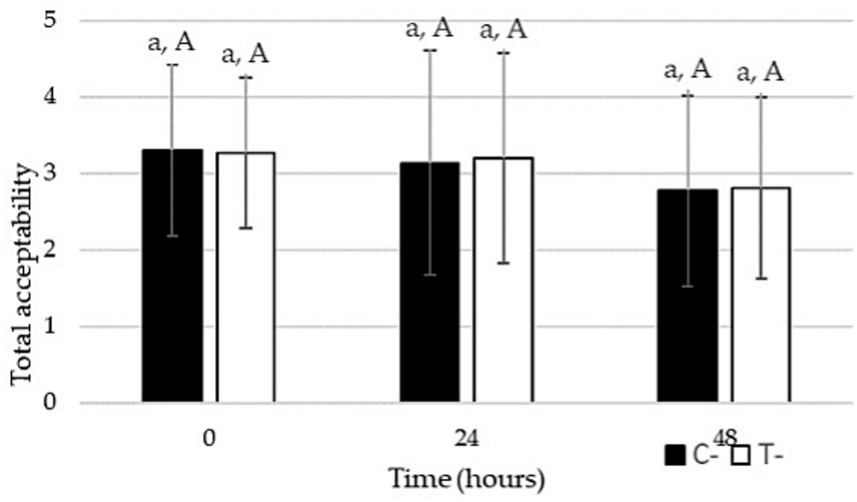
Total acceptability of rocket salad stored at 4 °C for 48 h. Significant differences (*p* < 0.05) for different samples at the same time are represented by different lowercase letters, whereas for the same sample at different times, by different uppercase letters.

Total acceptability did not differ significantly between C− and T− samples at T0, nor at T24, nor at T48. As shown in Figure 3, it decreased slightly over time due to the natural degradation the rocket undergoes, although, also in this case, not significantly. Finally, regarding consumers’ willingness to buy the sample they considered different, panelists expressed their intention to purchase 10 and 14 times out of 20, for C− and T− respectively at T0; 12 times out of 20 for both C− and T− at T24; and 15 and 14 times out of 20, for C− and T− respectively, at T48. So, in general, the willingness to buy was very similar between the two samples. This is an important result, underscoring that consumers can appreciate the treated rocket salad and that the sensory perception of the treatment fades over time.

## 4 Discussion

Hydrolates from various oregano species have been investigated for their *in vitro* antimicrobial activity, also against *L. monocytogenes*, giving promising results. However, the effectiveness was lower than that of the corresponding EOs, generally active at very low concentrations (Saǧdιç, 2003; Gülmez et al., 2006; Cid-Pérez et al., 2019; Gavriil et al., 2021). In the present study, a thorough analysis of the antimicrobial effectiveness of OHY on three *L. monocytogenes* strains was performed by a proper evaluation of its *in vitro* activity and its application on rocket leaves. *In vitro* investigation revealed inhibitory potential at a concentration of 125 μL mL^−1^. This allowed not only to reduce cells’ multiplication by reducing their growth potential but also to extend their lag phase, probably due to cell stress. More importantly, a 500 μL mL^−1^ OHY concentration completely inhibited wild-type *L. monocytogenes* strains and had a strong effect on *L. monocytogenes* ATCC 7644. Therefore, this concentration was used to evaluate OHY potential as a washing solution for rocket leaves. This strategy could be a way to exploit the hydrolate activity since this technique is not suitable for EOs. Promising antimicrobial results were obtained for OHY, particularly against the inoculated pathogen *L. monocytogenes*, and to a variable extent, against the microorganisms naturally occurring on rocket salad. Greater resistance to biopreservatives against spoilage, with respect to pathogenic microorganisms, has already been observed. The phenomenon could be explained by the fact that spoilage bacteria are normally exposed to natural compounds present in food. Thus, they could adapt to these substances, as the cytoplasmic membrane becomes less effective at preventing the entry of these molecules (Serio et al., 2014). This is suggested not only for *L. monocytogenes* but also for a wide range of pathogenic microorganisms that show a higher sensitivity to different hydrolases (Yavuzer and Kuley, 2020). The reduction of the inoculated *L. monocytogenes* was about 1 log CFU g^−1^, whereas the lowering of growth potential achieved by OHY showed that rocket remained a suitable matrix for the growth of *L. monocytogenes* even after the hydrolate treatment, although capable of reducing its load. This evidence is in agreement with Alegbeleye and Sant’Ana (2022), who demonstrated that leafy vegetables are a perfect substrate for *L. monocytogenes*, even under the refrigerated conditions in which they are normally stored. In fact, *L. monocytogenes* strains can persist for years in food processing facilities, where temperatures are maintained between 10 and 12 °C, leading to recurrent cross-contaminations in different products. This characteristic, as well as the presence of Stress Survival Islet (SSI) in the *L. monocytogenes* genome (Maggio et al., 2021), underlines the importance of exploring new strategies to reduce their presence and growth potential in food products. In fact, while the observed reduction in *L. monocytogenes* might be considered limited from regulatory or industrial perspectives, this result can fit perfectly within a multiple-hurdle context. In fact, rather than a single treatment, the use of OHY can also be seen as one hurdle in a multi-targeted technological strategy (Singh and Shalini, 2016).

*Pseudomonas* spp., Enterobacteriaceae, yeasts, and molds did not show relevant sensitivity against OHY. Instead, moderate activity was observed against *Bacillus* spp. and LAB, especially at T24. This result agrees with evidence according to which Gram-negatives, due to the outer lipopolysaccharide membrane, are generally more resistant to biopreservatives than Gram-positives (Martucci et al., 2015). Moreover, there was a slight impact on TMC and TPC, particularly in the non-inoculated samples, where the absence of *L. monocytogenes*, which is competitive at refrigeration temperatures, may have amplified the effect of OHY. It should be acknowledged as a study limitation that the high load of microbiota (~10^5^–10^6^ CFU g^−1^) of fresh rocket salad may have partially hindered the antimicrobial activity of OHY against *L. monocytogenes* as a possible consequence of the potential biotransformation of carvacrol into derivatives with reduced effectiveness, such as in the case of the fungal species naturally occurring on fresh produce (Mączka et al., 2023).

There is very limited data in the literature about the use of *Origanum* spp. hydrolates for *in situ* applications on leafy vegetables. Ozturk et al. (2016) tested the application of several HYs, including *Origanum onites*, on fresh-cut iceberg lettuce, inoculated with *L. monocytogenes* and other foodborne pathogens (Ozturk et al., 2016). Oregano was among the most effective hydrolates against *L. monocytogenes*, *Escherichia coli* O157: H7, and *Salmonella Typhimurium*, together with *Thymus vulgaris* and *Satureja hortensis* HYs. Oregano HY showed a reduction in *L. monocytogenes* load by around 2 log CFU g^−1^, with greater effectiveness at longer treatment times, which were between 20 and 50 min, than 5 min of dipping applied in our study. As a matter of fact, our treatment could be more exploitable at the industrial level due to the shorter processing time. Moreover, the reduction in microbial load was only assessed immediately after treatment with hydrolates, and no results were reported regarding the sensory impact of the treatment on the products (Ozturk et al., 2016). The main compound in *Origanum onites* was carvacrol (Ozturk et al., 2016), which is the most commonly present phenolic compound in oregano hydrolates (Bairamis et al., 2024, Kaplan et al., 2019, Mondal et al., 2021, Taglienti et al., 2022, Xylia et al., 2022). It is generally the main responsible for oregano activity, as confirmed by evidence of its antimicrobial properties, also *in situ* (Kraśniewska et al., 2020). Although highly effective, pure carvacrol cannot be used in food products because of its intense sensory notes, including a strong bitter taste and a stinging smell. However, it is plausible that the observed inhibitory activity cannot be attributed solely to the volatile compounds identified, as other polar molecules present in the hydrolate may have contributed to its overall antimicrobial effectiveness.

Nevertheless, other studies tested oregano hydrolates for the decontamination of various vegetable or non-vegetable matrices. Saǧdιç et al. (2013) and Törnük and Dertli (2014) reported the effectiveness of oregano hydrolates against foodborne pathogens (*E. coli* O157: H7 and *Staphylococcus aureus*) in tomatoes and cucumbers, and fresh-cut parsley, respectively. Xylia et al. (2022) demonstrated the efficacy of *Origanum dubium* HY and EO as dipping solutions for tomatoes and cucumbers against inoculated *L. monocytogenes*. The effectiveness was greater against this Gram-positive pathogen than against the Gram-negative *S. enterica*, consistent with the previously stated greater sensitivity of Gram-positive bacteria (Alegbeleye and Sant’Ana, 2022). When applied at the same concentration used in this study (500 μL mL^−1^), *O. dubium* hydrolate showed a reduction in *L. monocytogenes* comparable to our results at T0, whereas it exceeded 1 log CFU g^−1^ at day 7, compared to the control. Nevertheless, it should be taken into account that the treatment time was higher than that used in the present study (20 min against 5 min in our study) and thus, as previously evidenced, less competitive at an industrial level. However, longer treatment times and different doses could also be tested for OHY in rocket salad. Another documented food application of oregano hydrolate is its use as a dipping solution for decontaminating onion seeds, resulting in a lower incidence of molds (Rosińska, 2022). *O. vulgare* hydrolate was also successfully used for fungal decontaminations of papaya fruit (Culmone et al., 2023). Conversely, we did not observe a significant reduction in yeasts and molds, nor with OHY; nevertheless, these microbial groups are not particularly determinant of the shelf life of RTE rocket salad, despite post-harvest losses caused by fruit. Nonetheless, other studies suggest the *in situ* effectiveness of oregano hydrolate, especially when applied using the multiple hurdles approach (Willett et al., 2019). Indeed, the application of OHY to rocket salad could also be combined with other antimicrobial substances or modified-atmosphere packaging to increase its effectiveness. Moreover, different application techniques could be evaluated, although dipping is widely recognized as one of the best methods to allow a thorough, even distribution on the surfaces of vegetables (Mahmud and Khan, 2018). Future studies in this direction are desirable.

Oregano EOs, from whose processing the corresponding HYs derive, have been tested for their antimicrobial activity more extensively than HYs, but the strong sensory notes seem to limit their use in foods. For example, in the study by Gutierrez et al. (2008), oregano EO was successfully used to decontaminate RTE lettuce, but the panelists judged the treated samples as sensorially unacceptable. These observations show that EOs are rarely used on leafy vegetables, especially those with neutral flavors, such as lettuce, due to their penetrating aroma. Instead, hydrolates, much more delicate, could be a valid alternative due to their lower content in monoterpene hydrocarbon compounds, which make them less persistent in the food matrix (Tomi et al., 2016). Indeed, in the present study, OHY was generally positively perceived by consumers. In fact, as shown by the Triangle Test, consumer judgments, and the total acceptability test, the treated sample was recognized as significantly different from the control only at T0, as the oregano flavor declined over time. Furthermore, by developing the process at an industrial scale, a fresh-cut vegetable with compatible oregano sensory notes could be designed. In this way, hydrolates may positively contribute to the flavor of these products (Ozturk et al., 2016). This could also be achieved because rocket, compared to lettuce, has a naturally more aromatic and herbaceous flavor that could be compatible with that of HYs. In accordance with our findings, various studies showed a good sensory acceptance of oregano hydrolates when applied to vegetables (Ozturk et al., 2016, Saǧdιç et al., 2013). Xylia et al. (2022) also showed that the panelists considered tomatoes and cucumbers treated with *O. dubium* EO unacceptable, whereas the corresponding hydrolate presented better scores. However, in our study, the sensory notes of the biopreservatives naturally decreased over time.

Furthermore, the application of *O. dubium* HY to cucumbers showed greater tendency toward green color over time (Xylia et al., 2022). This result agrees with our findings, as OHY seemed to exert a protective effect on the green color (a* value), opposed to the red tendency, which indicates browning phenomena (Obulesu and Bhattacharya, 2011). Instead, in the present study, the effect of OHY on L* appeared less positive, which increased over time, suggesting color alteration of the leaves. This may indicate increased respiration rate and ethylene production, resulting in nutrient loss and chlorophyll degradation to colorless compounds (Ben-Fadhel et al., 2016, Veloso et al., 2019), and was observed similarly for both C− and T− samples. This phenomenon was already observed in lettuce (Peng et al., 2014). Moreover, b* values were significantly higher for T− than C− at T48, indicating a higher tendency to yellowing of the leaves. Values of ΔE indicated a greater similarity in global color between C− and T− samples at T48, at which time nearly all parameters equilibrated. Although many color quality parameters decreased over time, OHY treatment did not determine a worsening compared to the control; indeed, for the parameter a* (tendency toward green), a positive effect was observed. Furthermore, the judgments of the panelists revealed no differences between the two samples, except in one case in which the control was defined as lighter at T48 and therefore probably more faded.

The pH and aw values were within the normal range for both control and treated samples, with values comparable to those reported in the literature (Culliney and Schmalenberger, 2020, Zhang et al., 2020).

The composition of the hydrolate analyzed is consistent with results reported by other authors for *O. vulgare* L. hydrolates, with high percentages of carvacrol, followed by thymol (Khan, 2020). These two molecules exert biological activity against microorganisms, as well as linalool and terpinene-4-ol, as detected in our hydrolate, can impair the membrane integrity and permeability (Guo et al., 2021; Liu et al., 2026).

## 5 Conclusion

The present study provided a starting point for optimizing OHY treatment as a natural and innovative washing solution for the processing of fresh-cut rocket salad and for domestic washing, as an alternative to the sanitizing synthetic agents normally used for these products. Moreover, *O. vulgare* is widespread in the Mediterranean area, and the derived hydrolate could easily be used for this application, reducing the side effects of other washing treatments commonly used. Inoculated *L. monocytogenes*, a pathogen of particular concern for fresh-cut vegetables, showed reductions of nearly 1 log CFU g^−1^ in treated samples stored at 4 °C for 48 h, with the greatest effect observed at T24. Variable activity was observed against the spoilage microbial groups, which were more resistant than the pathogen; the greatest OHY activity was observed against total mesophilic and psychrophilic populations for uninoculated samples, indicating that *L. monocytogenes* may hinder the effect of the hydrolase in refrigeration storage. Interestingly, physical–chemical and sensory characteristics were not significantly affected, suggesting the suitability of hydrolate. Although the reduction of approximately 1 log CFU g^−1^ observed for *L. monocytogenes* indicates that OHY alone is not sufficient as a standalone sanitization strategy, it represents a promising starting point within a multiple-hurdle approach. Building on these encouraging results, it would be desirable to optimize OHY concentrations and times of treatment, also testing the combination with other biopreservatives in the formulation of the washing solution to boost the antimicrobial effect over time.

## Data Availability

The original contributions presented in the study are included in the article/supplementary material, further inquiries can be directed to the corresponding author.
